# A phase 1 open-label study to assess the relative bioavailability of TAK-931 tablets in reference to powder-in-capsule in patients with advanced solid tumors

**DOI:** 10.1007/s10637-022-01318-3

**Published:** 2022-11-21

**Authors:** Neeltje Steeghs, Melinda Pruis, Carla van Herpen, Vickie Lu, John Redman, Xiaofei Zhou

**Affiliations:** 1 The Netherlands Cancer Institute, Amsterdam, The Netherlands; 2 Erasmus MC Cancer Institute, Rotterdam, The Netherlands; 3 Radboud University Medical Center, Nijmegen, The Netherlands; 4 Takeda Development Center Americas, Inc. (TDCA), Cambridge, MA USA; 5 Proteus Ventures LLC, Kennett Square, PA USA

**Keywords:** Phase 1, TAK-931, CDC7 inhibitor, Bioavailability, Solid tumors

## Abstract

In this phase 1 open-label study, we assessed the relative bioavailability of a prototype tablet formulation of TAK-931, a cell division cycle 7 kinase inhibitor, in reference to the current powder-in-capsule (PIC) formulation in patients with advanced solid tumors for whom no effective standard treatment was available. Adult patients were randomized 1:1 in a crossover fashion to receive one dose of TAK-931 80 mg PIC on Day 1 and one dose of TAK-931 80 mg tablet on Day 3 (or the reverse sequence), followed by TAK-931 50 mg PIC once daily (QD) for 12 days starting from Day 5, before a 7-day rest period (Cycle 0). From Cycle 1, all patients received 50 mg PIC QD on Days 1–14 followed by a 7-day rest period. Twenty patients were enrolled. Median T_max_ was achieved approximately 2 h post-dose of TAK-931 80 mg for both tablet and PIC. Geometric mean C_max_, AUC exposures, and T_1/2z_ of TAK-931 were similar for both formulations. Geometric mean C_max_, AUC_last_, and AUC_inf_ ratios were 0.936 (90% confidence interval [CI]: 0.808–1.084), 1.004 (90% CI: 0.899–1.120), and 1.007 (90% CI: 0.903–1.123), respectively, for TAK-931 tablet in reference to PIC. Discontinuation of TAK-931 due to treatment-emergent adverse events (TEAEs) occurred in 1 patient. Four (20%) patients experienced a serious TEAE; none were considered related to TAK-931. Pharmacokinetics and systemic exposure profiles were similar following administration of both formulations, supporting the transition from PIC to tablet in the clinical development of TAK-931. (*Trial registration number* *ClinicalTrials.gov NCT03708211. Registration date October 12, 2018*).

**Supplementary Information:**

The online version contains supplementary material available at 10.1007/s10637-022-01318-3.

## Introduction

Cell division cycle 7 (CDC7) is a highly conserved serine/threonine kinase that has an essential role in initiation of DNA replication during replication stress [1–4], and is usually undetectable or low in healthy tissue [5]. CDC7 overexpression has been reported in several tumor types including ovarian, breast, lung, and oral cancers, as well as in diffuse large B-cell lymphoma [2, 5–7]. Increased CDC7 expression is also correlated with poor prognosis [1]. Thus, CDC7 could represent a novel therapeutic target and is an active area of research in cancer therapy [8–11].

TAK-931 is a highly potent and selective CDC7 kinase inhibitor which is currently undergoing clinical evaluation for the treatment of various malignancies [10]. In preclinical in-vivo and in-vitro cancer models, CDC7 inhibition by TAK-931 resulted in prolonged replication stress and consequent mitotic aberrations, and proliferation inhibition; furthermore, TAK-931 showed antiproliferative activity in a broad range of cancer cell lines and multiple patient-derived xenograft models [10].

An oral, powder-in-capsule (PIC) formulation of TAK-931, evaluated in a first-in-human phase 1 study in patients with solid tumors (NCT02699749), demonstrated an acceptable safety profile and preliminary antitumor activity [12]. The PIC formulation was also investigated in a phase 2 study in patients with metastatic pancreatic cancer, metastatic colorectal cancer, and other advanced solid tumors (NCT03261947). Clinical development of TAK-931 was subsequently transitioned to a tablet formulation for manufacturing scalability. The prototype tablet was designed as an immediate-release formulation with dose strengths of 10, 25, and 80 mg. The 80 mg tablet is the highest dosage being evaluated in clinical studies. We conducted this phase 1 open-label study to assess the relative bioavailability of the tablet formulation of TAK-931 in reference to the PIC formulation, as well as the pharmacokinetics (PK), safety, and antitumor activity of TAK-931 in patients with locally advanced or metastatic solid tumors.

## Methods

### Patients

This study enrolled adult patients with histologically or cytologically confirmed, locally advanced or metastatic solid tumors for whom no effective standard treatment was available. Patients were required to have: an Eastern Cooperative Oncology Group performance status of 0 or 1; a left ventricular ejection fraction ≥ 50%, as measured by echocardiogram or multiple gated acquisition scan; and adequate bone marrow reserve, and renal and hepatic function (absolute neutrophil count ≥ 1.5 × 10^9^/L; platelet count ≥ 75 × 10^9^/L; hemoglobin ≥ 85 g/L; bilirubin ≤ 1.5 × upper limit of normal [ULN]; alanine transaminase or aspartate transaminases ≤ 3.0 × ULN [elevation of either up to 5 × ULN was permitted if ascribed to underlying liver metastases]; and serum creatinine < 1.5 × the institutional ULN or estimated [Cockcroft-Gault formula] creatinine clearance of ≥ 30 mL/min for patients with serum creatinine concentrations above institutional limits). Patients were excluded: if they had received treatment with systemic anticancer treatments or investigational products within 28 days before the first TAK-931 dose or 5 half-lives, whichever was shorter; if they required continuous use of proton pump inhibitors (PPIs) or histamine-2 receptor antagonists, or had received treatment with PPIs within 5 days before the first dose of study drug; if they had received treatment with clinically significant enzyme inducers, such as phenytoin, carbamazepine, enzalutamide, mitotane, ritonavir, rifampin, or St John's wort within 14 days before the first TAK-931 dose; had hypertension that was not controlled with standard treatment; or had a known gastrointestinal disease or procedure that could interfere with the absorption of TAK-931. Patients with brain metastases were eligible if there was no evidence of progression for at least 4 weeks after central nervous system-directed treatment, assessed by clinical examination and brain imaging (magnetic resonance imaging or computed tomography) during screening. Patients had to have a radiographically or clinically evaluable tumor, but measurable disease as defined by Response Evaluation Criteria in Solid Tumors (RECIST) version 1.1 was not required. Full inclusion and exclusion criteria are described in the Supplementary Materials.

The study was conducted in accordance with the protocol, the ethical principles that have their origin in the Declaration of Helsinki, in accordance with the International Conference on Harmonisation Good Clinical Practice standards and applicable regulatory requirements, and in compliance with the Institutional Review Board regulations stated in the Good Clinical Practice regulations and guidelines. All patients provided written informed consent. The trial was designed in accordance with US Food and Drug Administration (FDA) guidelines on bioavailability and bioequivalence studies [13], and is registered at ClinicalTrials.gov (NCT03708211).

### Study design and endpoints

The relative bioavailability of the tablet in reference to the PIC formulation was characterized in a crossover PK study design (Supplementary Fig. 1). Patients were randomized 1:1 to receive one dose of TAK-931 80 mg PIC on Day 1 of Cycle 0 followed by one dose of TAK-931 80 mg tablet on Day 3, or to receive the reverse sequence. TAK-931 was administered on an empty stomach, except for water, from 2 h before taking the study drug until completion of collection of the 4-h ECG/PK samples on Day 1 and Day 3 of Cycle 0. Blood samples were collected predose and up to 48 h postdose at predetermined time points to measure plasma drug concentrations In both schedules, TAK-931 50 mg PIC was then administered once daily (QD) for 12 days starting from Day 5, followed by a 7-day rest period in Cycle 0. From Cycle 1, all patients received 50 mg PIC QD on Days 1–14 followed by a 7-day rest period in 21-day treatment cycles. Patients continued treatment for 1 year or until they experienced disease progression, unacceptable toxicity, or any other discontinuation criteria were met.

The primary endpoints were the ratio of geometric means of the following PK parameters for TAK-931 tablets in reference to PIC and associated 2-sided 90% confidence intervals (CIs): maximum observed plasma concentration (C_max_), area under the plasma concentration–time curve (AUC) from time 0 to time of the last quantifiable concentration (AUC_last_), and the AUC from time 0 to infinity (AUC_inf_). Secondary endpoints were PK parameters of TAK-931 80 mg following a single-dose administration as PIC or tablet (time of first occurrence of maximum observed plasma concentration [T_max_], and terminal disposition phase half-life [T_1/2z_]), antitumor activity, and safety.

### Assessments

Blood samples for PK analysis were collected within 1 h before dosing and at 30 min, and 1, 2, 4, 6, 8, 24, and 48 h post-dose in Cycle 0, Days 1 and 3. Plasma concentrations of TAK-931 were determined using a validated liquid chromatography tandem mass spectrometry method [14], with a dynamic range of 0.5 to 500 ng/mL. Toxicity was evaluated according to National Cancer Institute Common Terminology Criteria for Adverse Events, version 5.0, and disease response was assessed by the investigator according to RECIST version 1.1.

### Statistical analyses and sample size

Relative bioavailability analysis of the tablet formulation relative to the PIC formulation was performed by an analysis of variance on C_max_ and AUC_0-last_/AUC_0-inf_. The relative bioavailability of the tablet versus PIC formulation was estimated as the ratio of geometric mean values of C_max_ and AUC_0–last_/AUC_0-inf_, (tablet:PIC), and associated 2-sided 90% CI. TAK-931 PK parameters were estimated using noncompartmental methods with WinNonlin Phoenix version 6.2 (Pharsight Corporation, Mountain View, CA, USA). Descriptive statistics for TAK-931 plasma concentrations and PK parameters are reported by formulation.

It was calculated that approximately 14–16 PK-evaluable patients were required; the sample size calculation was based on the expected 2-sided 90% CI for the difference in the paired, log-transformed AUC (or C_max_) means on Days 1 and 3. According to preliminary data obtained from study NCT02699749, the within-patient coefficient of variation was estimated to be 35.0% for AUC and 36.9% for C_max_, respectively. Assuming the AUC ratio of the two formulations (tablet vs PIC) was 1, with a sample size of 14, the 90% CI for the AUC ratio was expected to be (0.795–1.257) for AUC and (0.786–1.272) for C_max_. Patients who were not PK-evaluable may have been replaced to ensure the availability of 14–16 PK-evaluable patients in the final analysis. Assuming that up to 4–6 patients would need to be replaced, approximately 20 patients were planned for enrollment.

## Results

### Patients

A total of 20 patients were randomized to receive TAK-931; of these, 8 received the tablet formulation first and 12 received the PIC formulation first in Cycle 0. All patients discontinued treatment: 16 due to progressive disease (PD), 2 due to symptomatic deterioration, and 2 for unspecified reasons. Baseline demographics and disease characteristics are described in Table 1. Overall, median age was 63 years (range 43–84) and 12 (60%) patients were male. The most common diagnoses were colon cancer (n = 5), and non–small cell lung cancer and rectal cancer (n = 2 each); all other cancer types occurred in 1 patient each. All patients had received prior therapy, with 8 (40%) patients receiving ≥ 4 previous lines of therapy.

**Table 1:** Patient demographics and disease characteristics

	Tablet/PIC	PIC/Tablet	Total
	n = 8	n = 12	N = 20
Median age, years (range)	54 (44–84)	64 (43–77)	63 (43–84)
Male, n (%)	4 (50)	8 (67)	12 (60)
Disease at diagnosis, n (%)			
Colon cancer	1 (13)	4 (33)	5 (25)
Non-small cell lung cancer	0	2 (17)	2 (10)
Rectal cancer	1 (13)	1 (8)	2 (10)
Other*	6 (75)	5 (42)	11 (55)
Disease stage at study entry, n (%)			
III	2 (25)	2 (17)	4 (20)
IV	6 (75)	10 (83)	16 (80)
ECOG PS, n (%)			
0	4 (50)	9 (75)	13 (65)
1	4 (50)	3 (25)	7 (35)
Prior lines of therapy, n (%)			
1	1 (13)	1 (8)	2 (10)
2	2 (25)	4 (33)	6 (30)
3	1 (13)	3 (25)	4 (20)
≥ 4	4 (50)	4 (33)	8 (40)

*ECOG PS* Eastern Cooperative Oncology Group Performance Status, *ER* estrogen receptor, *HER2* human epidermal growth factor receptor 2, *PIC* powder-in-capsule, *PR* progesterone receptor

^*^Diagnoses in 1 patient only: Tablet/PIC: HER2/neu-positive and ER- or PR- positive breast cancer, cholangiocarcinoma, clear cell renal carcinoma, malignant pleural mesothelioma, metastatic squamous neck cancer with occult primary, and testicular cancer; PIC/Tablet: nasopharyngeal carcinoma, neuro-endocrine carcinoma of the esophagus, penile cancer, urachal cancer, vulvar cancer

### Relative bioavailability

**Fig. 1:**
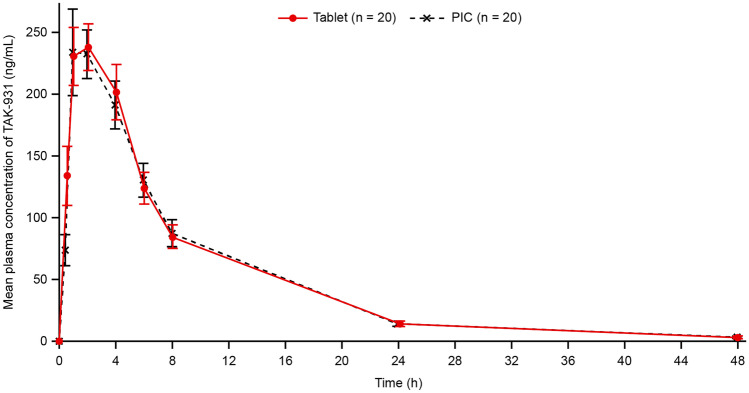
Mean (and standard deviation) plasma concentration–time profiles of TAK-931 following oral administration of PIC or tablet at 80 mg. *h* hours, *h* PIC, powder in capsule

All patients (N = 20) were included in the assessment of relative bioavailability of TAK-931 tablet in reference to PIC. Mean TAK-931 plasma concentration–time profiles following single-dose oral administration at 80 mg as tablet or PIC are shown in Fig. 1. A summary of key PK parameters is shown in Table 2. Following single oral-dose administration of TAK-931 at 80 mg, median T_max_ was achieved approximately 2 h post-dose for both tablet and PIC. The geometric mean C_max_ and AUC exposures of TAK-931 were similar following administration as tablet and PIC. T_1/2z_ was also similar (7.5 h) following oral dosing as tablet or PIC. Inter-patient variability in systemic exposure (coefficient of variation) was comparable with tablet and PIC formulations. Individual comparisons of C_max_ and AUC_inf_ following administration as tablet or PIC are shown in Supplementary Fig. 2.

**Table 2:** Summary of key TAK-931 PK parameters following single dose administration as a tablet or PIC and statistical analysis for estimation of relative bioavailability (tablet in reference to PIC)

	**C**_**max**_** (ng/mL)**		**AUC**_**last**_** (hr·ng/mL)**		**AUC**_**inf**_** (hr·ng/mL)**		**T**_**max**_** (hr)**	**T**_**1/2z**_** (hr)**
	**Geometric mean (CV%)**		**Geometric mean (CV%)**		**Geometric mean (CV%)**		**Median** **(min–max)**	**Mean** **(std dev)**
Tablet	270.1 (31%)		1,899 (42%)		1,926 (42%)		1.98 (0.48–4.0)	7.5 (1.2)
PIC	251.5 (48%)		1,869 (48%)		1,901 (48%)		1.68 (0.92–4.1)	7.5 (1.7)

	**LS mean**	**GMR** (90% CI)*	**LS mean**	**GMR** (90% CI)*	**LS mean**	**GMR** (90% CI)*		
Tablet	268.4	0.936 (0.808–1.084)	1,875	1.004 (0.899–1.120)	1,901	1.007 (0.903–1.123)		
PIC	251.2		1,882		1,914			

N = 20

*CV%* percent coefficient of variation, *AUC*_*inf*_ area under the concentration–time curve from time 0 to infinity, calculated using the observed value of the last quantifiable concentration, *AUC*_*last*_ area under the concentration–time curve from time 0 to time of the last quantifiable concentration; *CI* confidence interval, *C*_*max*_ maximum observed plasma concentration, *GMR* geometric mean ratio tablet:PIC, *hr* hours, *LS* least squares, *max* maximum, *min* minimum, *PIC* powder-in-capsule, *PK* pharmacokinetic, std *dev* standard deviation, *T*_*1/2z*_ terminal disposition phase half-life, *T*_*max*_ time of first occurrence of C_max_

^*^CIs were calculated for the difference in the LS means of the natural log–transformed AUC_last_, AUC_inf_, or C_max_ values (difference = tablet/PIC). Antilogs of the confidence limits for the difference were taken to construct the CIs for the ratio of the geometric means. All parameters presented are in the original scale

The statistical analyses of TAK-931 exposures for the estimation of relative bioavailability of TAK-931 as tablet in reference to PIC are shown in Table 2. Geometric mean C_max_ and AUC exposures of TAK-931 were similar following administration as tablet or PIC. The geometric mean C_max_ ratio was 0.936 with an associated 90% CI of (0.808–1.084) for TAK-931 tablet in reference to PIC. The geometric mean AUC_last_ ratio was approximately 1 with a 90% CI of (0.899–1.120) for TAK-931 tablet versus PIC. The geometric mean AUC_inf_ ratio was approximately 1 with a 90% CI of (0.903–1.123) for TAK-931 tablet in reference to PIC.

### Safety and antitumor activity

All patients received at least one dose of study drug and were included in the safety analysis, summarized in Table 3. Most patients completed at least three treatment cycles, with a median number of doses of 42 and a median dose intensity of 100%. Overall, 10 (50%) patients had at least one dose modification, including 3 (15%) patients whose dose was reduced. The most common reason for dose modification, in 7 (35%) patients, was treatment-emergent adverse events (TEAEs). A total of 19 (95%) patients experienced at least one TEAE; the most common included fatigue, (65%), constipation, (40%), nausea (40%), vomiting (30%), and alopecia (30%) (Table 4). Overall, 8 (40%) patients experienced at least one grade ≥ 3 TEAE; the most common were neutropenia and ileus (each reported in 10% of patients). One patient with rectal cancer died due to PD; this was not considered related to treatment with TAK-931. Seventeen (85%) patients experienced at least one treatment-related TEAE, the most common being fatigue (50%), alopecia (30%), nausea (30%), and vomiting and decreased appetite (both 20%). Two (10%) patients reported at least one grade ≥ 3 treatment-related TEAE; 1 patient with neutropenia, and the other with neutropenia and hypertension. One patient experienced three TEAEs (aphasia, headache, and disturbance in attention) resulting in TAK-931 discontinuation. Four (20%) patients experienced a serious TEAE, but none of the reported events were considered related to TAK-931.

**Table 3:** Safety and tolerability summary

	Tablet/PIC	PIC/Tablet	Total
n (%)	n = 8	n = 12	N = 20
**TEAEs**	8 (100)	11 (92)	19 (95)
Related	7 (88)	10 (83)	17 (85)
Not related	8 (100)	10 (83)	18 (90)
Grade ≥ 3	3 (38)	5 (42)	8 (40)
Grade ≥ 3 related	1 (13)	1 (8)	2 (10)
Leading to TAK-931 discontinuation	0	1 (8)	1 (5)
**Serious TEAEs**	2 (25)	2 (17)	4 (20)
Related	0	0	0
Not related	2 (25)	2 (17)	4 (20)
**On-study deaths**	1 (13)	0	1 (5)
**Dose modification**	6 (75)	4 (33)	10 (50)
Dose increased	0	0	0
Dose reduced	3 (38)	0	3 (15)
Treatment withdrawn	0	1 (8)	1 (5)
Dose delayed	3 (38)	4 (33)	7 (35)
Dose missed	1 (13)	0	1 (5)
**Reason for dose modification**	6 (75)	4 (33)	10 (50)
AE	4 (50)	3 (25)	7 (35)
Other	3 (38)	2 (17)	5 (25)

*AE* adverse event*, PIC* powder-in-capsule, *TEAE* treatment-emergent adverse event

**Table 4:** Most common TEAEs

	N = 20	
n (%)	Any-grade	Grade ≥ 3
Fatigue	13 (65)	1 (5)
Constipation	8 (40)	-
Nausea	8 (40)	-
Vomiting	6 (30)	-
Alopecia	6 (30)	-
Decreased appetite	4 (20)	-
Hypertension	3 (15)	1 (5)
Headache	3 (15)	-
Weight decrease	3 (15)	-
Dyspnea	3 (15)	1 (5)
Disturbance in attention	2 (10)	-
Ileus	2 (10)	2 (10)
Neutropenia	2 (10)	2 (10)
Pneumonia	1 (5)	1 (5)
Hypokalemia	1 (5)	1 (5)
Rectal cancer	1 (5)	1 (5)

TEAEs shown by preferred term in all treatment schedules

*TEAE* treatment-emergent adverse event

A total of 19 patients were evaluable for response. No partial or complete responses to TAK-931 were seen. Four (21%) patients had a best response of stable disease (SD), with individual progression-free survival durations of 3.58, 2.23 + (censored observation), 3.55, and 4.01 months. Median time to PD or death was 1.9 months (95% CI: 1.6–2.8).

## Discussion

An oral PIC formulation of the CDC7-selective small molecule inhibitor, TAK-931, has been evaluated in phase 1/2 clinical trials (NCT02699749 [12] and NCT03261947). To facilitate manufacture scale-up and enable the transition from PIC in clinical development, a tablet formulation of TAK-931 80 mg has been developed. The purpose of this phase 1 open-label study, designed based on FDA guidelines [13], was to characterize the relative bioavailability of TAK-931 administered as a prototype tablet in reference to the existing PIC formulation. PK, safety, and antitumor activity of TAK-931 were also assessed.

Considering the potential mechanisms of action of TAK-931 (interfering with DNA repair and replication, and subsequently inducing apoptosis in cancer cell lines [10]), this study was conducted in patients with locally advanced or metastatic solid tumors. The crossover study design allowed for patients to be their own control, resulting in robust comparable measurements between the tablet and PIC formulations. Notably, the relative bioavailability data generated in this study showed that the PKs and systemic exposures of TAK-931 were similar following administration of the two formulations.

The safety analysis indicated that treatment with TAK-931 was well tolerated, with an acceptable toxicity profile. TEAEs were most commonly gastrointestinal in nature, consistent with those previously reported in the first-in-human phase 1 study [12] and the phase 2 study (NCT03261947). As the relative bioavailability of the tablet was similar in reference to PIC, the safety profile of the tablet formulation would be expected to be similar to the PIC formulation. However, this requires further validation, as participants only received one dose of the tablet formulation in this study.

TAK-931 activity was not evident in this cohort of patients with locally advanced or metastatic solid tumors. These results are not consistent with the antitumor activity reported in the first-in-human phase 1 study, in which 4 of 24 response-evaluable patients had prolonged SD and 3 experienced a partial response [12]. Further data are required to better elucidate the potential clinical activity of TAK-931 in this patient population.

In conclusion, the PKs and systemic exposures of TAK-931 were similar following administration of the tablet and PIC formulations. The PK and safety results support the transition from PIC to the tablet formulation, which will facilitate the clinical development of TAK-931.

## Supplementary Information

Below is the link to the electronic supplementary material.

Supplementary file1 (PDF 571 KB)

## Data Availability

The datasets, including the redacted study protocol, redacted statistical analysis plan, and individual participants’ data supporting the results reported in this article, will be made available within 3 months from initial request to researchers who provide a methodologically sound proposal. The data will be provided after its de-identification, in compliance with applicable privacy laws, data protection, and requirements for consent and anonymization.
