# Supplementary material for: A phase 1 open-label study to assess the relative bioavailability of TAK-931 tablets in reference to powder-in-capsule in patients with advanced solid tumors
Source: Invest New Drugs. 2022 Nov 21;41(1):53–9. doi: 10.1007/s10637-022-01318-3 (PMC10030390; doi:10.1007/s10637-022-01318-3)
Supplement: Supplementary file 1 — Supplementary file1 (PDF 571 KB) [file 10637_2022_1318_MOESM1_ESM.pdf]

Supplementary materials

Journal: *Investigational New Drugs*

A phase 1 open-label study to assess the relative bioavailability of TAK-931 tablets in reference to powder-in-capsule in patients with advanced solid tumors

Neeltje Steeghs[1] · Melinda Pruis[2] · Carla Van Herpen[3] · Vickie Lu[4] · John Redman[5] · Xiaofei Zhou[4]

Affiliations:

[1]The Netherlands Cancer Institute, Amsterdam, The Netherlands

[2]Erasmus MC Cancer Institute, Rotterdam, The Netherlands

[3]Radboud University Medical Center, Nijmegen, The Netherlands

[4]Takeda Development Center Americas, Inc., Lexington, MA, USA

[5]Proteus Ventures LLC, Kennett Square, , PA, USA

Corresponding author: Xiaofei Zhou

Email: Xiaofei.Zhou@Takeda.com

Full inclusion criteria

Each patient was required to meet all the following inclusion criteria to be enrolled in the study:

1. Male or female patients aged 18 years or older.
2. Adult patients with histologically or cytologically confirmed metastatic or locally advanced solid tumors for whom there was no available standard treatment with proven survival benefit, this therapy was not indicated, or it was refused by the patient. Based on nonclinical data, the following indications may have had a higher probability of clinical benefit: high-grade serous ovarian cancer, uterine carcinosarcoma, squamous esophageal cancer, squamous non–small cell lung cancer, squamous head and neck cancers, rectal adenocarcinoma, and in general tumors with known *TP53* gene mutations. For any of these preferred indications, patients should have exhausted standard therapeutic options with a proven survival benefit.
3. Eastern Cooperative Oncology Group performance status of 0 to 1.
4. Adequate bone marrow reserve, and renal and hepatic function based on the following laboratory parameters:
   - Absolute neutrophil count ≥ 1.5 × 10^9/L, platelet count ≥ 75 × 10^9/L, and hemoglobin ≥ 85 g/L.
   - Total bilirubin ≤ 1.5 times the institutional upper limit of the normal range (ULN) or total bilirubin < 3.0 times the ULN in patients with well-documented Gilbert syndrome.
   - Serum alanine aminotransferase or aspartate aminotransferase ≤ 3.0 × ULN (< 5 × ULN if liver enzyme elevations were due to hepatocellular cancer, biliary tract cancer, or metastatic disease in the liver).

- Creatinine < 1.5 × the institutional ULN or estimated creatinine clearance (using the Cockcroft-Gault formula) ≥ 30 mL/min for patients with serum creatinine concentrations above institutional limits.

5. Left ventricular ejection fraction ≥ 50%, as measured by echocardiogram or multiple-gated acquisition scan, within 4 weeks before receiving the first dose of study drug.
6. Recovered to grade 1 or baseline from all toxic effects of previous therapy (except alopecia or neuropathy).
7. Female patients who:
    - Were postmenopausal for at least 1 year before the screening visit, OR
    - Were surgically sterile, OR
    - If they were of childbearing potential, had agreed to practice one highly effective method of contraception and one additional effective (barrier) method at the same time, from the time of signing the informed consent form through 30 days after the last dose of study drug, OR
    - Had agreed to practice true abstinence, when this was in line with the preferred and usual lifestyle of the patient. (Periodic abstinence [e.g., calendar, ovulation, symptothermal, post-ovulation methods], withdrawal, spermicides only, and lactational amenorrhea were not acceptable methods of contraception. Female and male condoms were not to be used together).
8. Male patients, even if surgically sterilized (i.e., status post-vasectomy), who:
    - Had agreed to practice effective barrier contraception during the entire study treatment period and through 120 days after the last dose of study drug, OR
    - Had agreed to practice true abstinence, when this was in line with the preferred and usual lifestyle of the patient. (Periodic abstinence [e.g.,

calendar, ovulation, symptothermal, post-ovulation methods], withdrawal, spermicides only, and lactational amenorrhea were not acceptable methods of contraception. Female and male condoms were not to be used together), AND

- Had agreed not to donate sperm during this study and for 120 days after receiving their last dose of study drug.

9. Voluntary written consent was given before performance of any study-related procedure not part of standard medical care, with the understanding that consent could be withdrawn by the patient at any time without prejudice to future medical care.
10. Suitable venous access for the study-required blood sampling including pharmacokinetic sampling.
11. Patients had a radiographically or clinically evaluable tumor, but measurable disease as defined by Response Evaluation Criteria in Solid Tumors (version 1.1) was not required for participation in this study.

Full exclusion criteria

1. Female patients who were lactating and breastfeeding or had a positive serum pregnancy test during the screening period or a positive urine pregnancy test on Day 1 before the first dose of study drug.
2. Treatment with systemic anticancer treatments or investigational products within 28 days before the first dose of study drug or 5 half-lives, whichever was shorter.
3. Patients who required continuous use of proton pump inhibitors (PPIs) or histamine-2 receptor antagonists, and patients who were taking PPIs within 5 days before the first dose of study drug.

4. Treatment with clinically significant enzyme inducers, such as phenytoin, carbamazepine, enzalutamide, mitotane, ritonavir, rifampin, or St John's wort within 14 days before the first dose of study drug.
5. Patients with hypertension that was unstable or not controlled despite appropriate medical therapy.
6. Patients with treated brain metastases were eligible if there was no evidence of progression for at least 4 weeks after central nervous system-directed treatment, as ascertained by clinical examination and brain imaging (magnetic resonance imaging or computed tomography) during the screening period.
7. Known history of human immunodeficiency virus infection.
8. Known hepatitis B virus surface antigen seropositive or detectable hepatitis C virus infection viral load. Note: patients who had positive hepatitis B core antibody or hepatitis B surface antigen antibody could have been enrolled but must have had an undetectable hepatitis B virus viral load.
9. Known gastrointestinal (GI) disease or GI procedure that could interfere with the GI absorption of study drug, such as total gastrectomy or GI conditions that could substantially modify gastric pH or GI transit.
10. Any serious medical or psychiatric illness that could, in the investigator’s opinion, potentially interfere with the completion of treatment according to this protocol.

Supplementary figures

Supplementary Fig. 1 Crossover study design

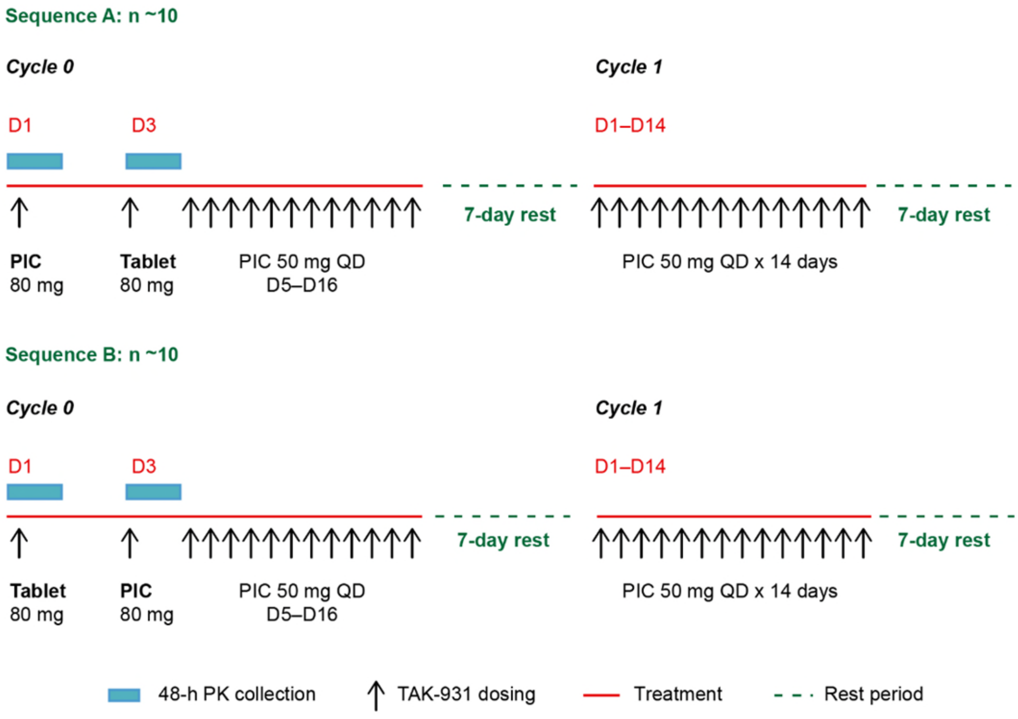

D day, *h* hour, *PK* pharmacokinetic, *QD* once daily, *PIC* powder-in-capsule

Supplementary Fig. 2 TAK-931 C_{max} (a) and AUC_{inf} (b) following oral administration of TAK-931 as tablet or PIC in individual patients

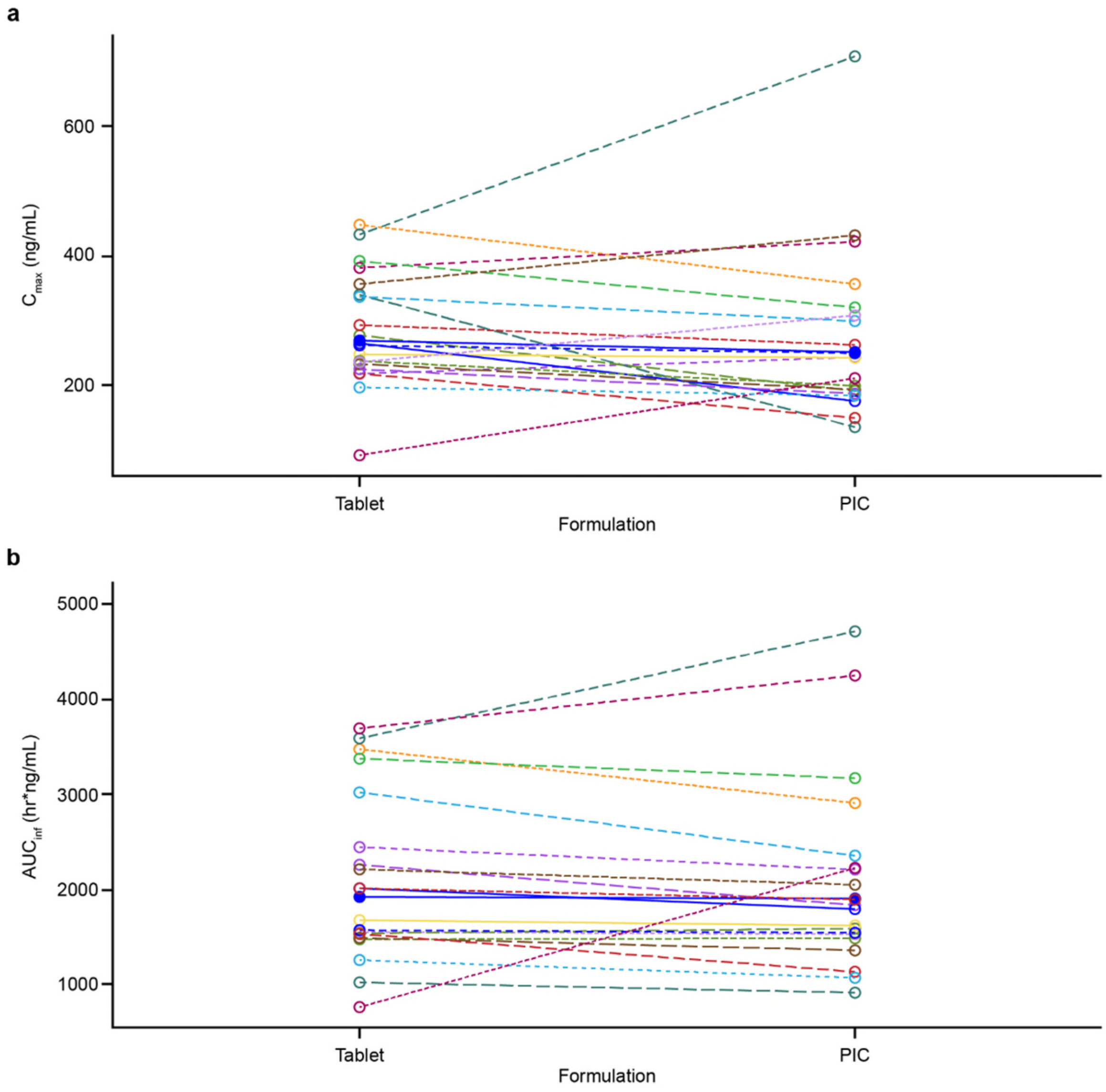

AUC_{inf} area under the concentration–time curve from time 0 to infinity, calculated using the observed value of the last quantifiable concentration, *C_{max}* maximum observed plasma concentration, *hr* hours, *PIC* powder-in-capsule
